# Bayesian alternatives to null hypothesis significance testing in biomedical research: a non-technical introduction to Bayesian inference with JASP

**DOI:** 10.1186/s12874-020-00980-6

**Published:** 2020-06-05

**Authors:** Riko Kelter

**Affiliations:** Department of Mathematics, University of Siegen, Walter-Flex-Str. 3, Siegen, 57072 Germany

**Keywords:** Bayesian hypothesis testing, Null hypothesis significance testing, JASP, Medical decision making, Replication crisis

## Abstract

**Background:**

Although null hypothesis significance testing (NHST) is the agreed gold standard in medical decision making and the most widespread inferential framework used in medical research, it has several drawbacks. Bayesian methods can complement or even replace frequentist NHST, but these methods have been underutilised mainly due to a lack of easy-to-use software. JASP is an open-source software for common operating systems, which has recently been developed to make Bayesian inference more accessible to researchers, including the most common tests, an intuitive graphical user interface and publication-ready output plots. This article provides a non-technical introduction to Bayesian hypothesis testing in JASP by comparing traditional tests and statistical methods with their Bayesian counterparts.

**Results:**

The comparison shows the strengths and limitations of JASP for frequentist NHST and Bayesian inference. Specifically, Bayesian hypothesis testing via Bayes factors can complement and even replace NHST in most situations in JASP. While *p*-values can only reject the null hypothesis, the Bayes factor can state evidence for both the null and the alternative hypothesis, making confirmation of hypotheses possible. Also, effect sizes can be precisely estimated in the Bayesian paradigm via JASP.

**Conclusions:**

Bayesian inference has not been widely used by now due to the dearth of accessible software. Medical decision making can be complemented by Bayesian hypothesis testing in JASP, providing richer information than single *p*-values and thus strengthening the credibility of an analysis. Through an easy point-and-click interface researchers used to other graphical statistical packages like SPSS can seemlessly transition to JASP and benefit from the listed advantages with only few limitations.

## Background

Null hypothesis significance testing (NHST) remains the dominating inferential approach in medical research [1–4]. The results of medical research therefore stand on the shoulders of the frequentist statistical philosophy, which roots back to the early days of Fisher [5] and Neyman-Pearson [6]. The centerpiece of frequentist inference is a test statistic *T*, which can be computed from the raw data, and which is known to have a specific distribution *F* under the null hypothesis *H*_0_. If the observed value of the test statistic passes a given threshold, which is located in the tails of *F*, then the null hypothesis *H*_0_ is rejected, because observing such a value would be quite unplausible if *H*_0_ were true. The well known *p*-value states exactly the probability of observing a result as extreme as the one observed or even more extreme when the null hypothesis *H*_0_ were true.

While the agreed standard in medical decision making, in the last few years more and more problems inherent in NHST have been revealed [7–9]. The misuse and abuse of *p*-values in particular in medical research have been criticised in countless venues, and the official American Statistical Association (ASA) statement in 2016 and 2019 by Wasserstein and Lazar [10] and Wasserstein et al. [11] show that most problems of NHST have not been solved by now. The ongoing use of NHST also indicates that the *p*-value as a measure of significance is still widely accepted despite its drawbacks and stays resilient to the repeated criticism [12]. As the limitations of *p*-values have been discussed widely, only three important problems are listed here, which are especially harmful in medical decision making and research: (1) it is known that *p*-values are prone to overestimating effects [13]; (2) they inevitably state effects if none exist with a fixed probability [14]; (3) they are prone to false interpretation by researchers [15]. This problem is in particular problematic in clinical decision making with possibly devastating consequences for patients and the progress of medical science, see Ioannidis [9, 16]. Especially point (2) is crucial, as not only for medical science but in much more generality, McElreath and Smaldino [17] stress that *“the most important factors in improving the reliability of research are the rate of false positives”*.

To solve the above problems inherent to NHST, researchers from the University of Amsterdam have developed the open-source statistical software JASP [18], which is an acronym for *Jeffreys Awesome Statistics Package*, referring to the pioneer of Bayesian statistics who invented the Bayes factor, Sir Harold Jeffreys [19]. JASP is available for all common operating systems and provides both frequentist NHST as well as Bayesian tests and methods. Installation is straightforward and there is rich documentation in form of tutorials and videos on the project website. A strength of JASP is its spreadsheet design similar to SPSS, making it possible to conduct state of the art analyses with a single click instead of programming complicated routines in statistical programming languages like R [20]. Also, to foster reproducible medical research, JASP offers seamless integration with the Open Science Framework [21] as well as shareable JASP-files which include all data and analyses, to promote collaboration and transparency. Next to this, JASP also benefits from rich annotations and information to enhance understanding of the applied procedures. To understand how JASP tackles the problems of NHST it is important to understand the differences of the proposed Bayesian methods, which are reviewed therefore briefly in the following.

NHST with its *p*-values is located in the frequentist school of statistics and was created to control the type I error rate in the long run, that is to limit the number of false positives in a large succession of repeatable experiments or studies. The Bayesian school of thought was not designed with type I error control in mind and proceeds via allocating relative evidence to a hypothesis *H* given the data *x* [22]. In the Bayesian paradigm, available prior information is combined with the model likelihood to obtain the posterior distribution of the parameters of interest [23]. Bayesian hypothesis testing is then often done via the Bayes factor *BF*_10_, the predictive updating factor which measures the change in relative beliefs about hypothesis *H*_1_ relative to hypothesis *H*_0_ given the data *x*:

1 $$ \begin{aligned} \underbrace{\frac{p\left(x|H_{1}\right)}{p\left(x|H_{0}\right)}}_{BF_{10}(x)} =\underbrace{\frac{\mathbb{P}\left(H_{1}|x\right)}{\mathbb{P}\left(H_{0}|x\right)}}_{\text{Posterior odds}} \cdot \underbrace{\frac{\mathbb{P}\left(H_{0}\right)}{\mathbb{P}\left(H_{1}\right)}}_{\text{Prior odds}} \end{aligned}  $$

The Bayes factor *BF*_10_ therefore quantifies the evidence by indicating how much more likely the observed data are under the rival models. Note that the Bayes factor critically depends on the prior distributions assigned to the parameters in each of the models, as the parameter values determine the models’ predictions. It can also be rewritten as the ratio of posterior and prior odds. Bayesian parameter estimation for an unknown parameter *θ* in general is achieved by considering the posterior distribution *p*(*θ*|*x*) of the parameter after observing the data *x*:

2 $$ \begin{aligned} p\left(\theta|x\right)=\frac{p\left(x|\theta\right)\cdot p\left(\theta\right)}{p(x)} \end{aligned}  $$

where *p*(*x*|*θ*) is the likelihood function, *p*(*θ*) the prior, and in most realistic settings, the marginal likelihood *p*(*x*) in the denumerator cannot be calculated in closed form or is prohibitively effortful to compute. Therefore, Markov-Chain-Monte-Carlo (MCMC) algorithms have been developed in the last decades, alleviating the requirement of computing *p*(*x*) from practitioners, because most MCMC algorithms only need a function *proportional* to the posterior to work, so that

3 $$ \begin{aligned} {p(\theta|x)\propto p(x|\theta)\cdot p(\theta)} \end{aligned}  $$

suffices. Equation (2) also implies, that specifying the prior *p*(*θ*) and likelihood *p*(*x*|*θ*) allows researchers to numerically obtain the posterior via MCMC.

In both the hypothesis testing as well as parameter estimation perspective in Bayesian inference, the role of the prior is crucial. The prior distribution quantifies the prior information about any parameters in the model *before* the data *x* are acutally observed. In contrast, the classical frequentist philosophy proceeds without any prior information, obtaining the same results no matter if there is much evidence in form of a large number of previous studies which all yielded identical results, or no evidence due to no available prior studies at all. While this may bring a subjective flavour with it, selecting an appropriate prior is a topic of huge relevance in Bayesian literature, as extreme priors can shrink the posterior estimates of a parameter or the obtained Bayes factor into a desired direction specified by the prior’s shape. Luckily, there is an unspoken agreement to use uninformative pri ors in most cases [22, 24], especially when no prior nformation is available (for example in form of results of pilot studies). This makes it easy to use a suitable prior in most standard tests and methods. For example, in medical research most often the effect size *d* of Cohen [25] is important. The effect size is used to quantify the effect of a treatment, or the effect between a treatment and control group, and a priori it is reasonable to assume that very large effects |*d*|>1 are less probable than small effects |*d*|≤1, as often in biomedical research small to medium effect sizes (0.2≤|*d*|<0.5) are observed. Common choices of prior distributions for the effect size are the normal distribution [26], t-distribution and the Cauchy distribution [27]. A common approach also includes to use uniform priors or priors with extremely large scale parameters like $\mathcal {N}(0,500)$ if no information is available for the parameter of interest [24]. It should be noted that this approach is problematic and should be avoided, as it can be shown that the a priori assumption then often degenerates to statements which believe much more probability mass in the tails as in the center of the distribution, essentially making the prior distributional assumption questionable. For example, a $\mathcal {N}(0,500)$ prior will tend to put much more probability mass on unreasonable parameter values than reasonable ones. To be more specific, this prior implies that one believes a priori that $\mathbb {P}(|\theta |<250)<\mathbb {P}(|\theta |>250)$, which is easily shown by calculating $\mathbb {P}(-250<\theta <250)\approx 0.38$. Even worse, pioneers of Bayesian inference like Jeffreys [27] already noticed that such unrealistic overdispersed priors can lead to situations in which the Bayes factor always signals evidence for the null hypothesis *H*_0_, even if the data *x* are indeed generated by the alternative *H*_1_. To prevent such problems, often slightly informative or weakly informative priors are used, which span a realistic range of values of the parameter a priori, but are not completely flat [28].

If a reasonable weakly informative prior is selected, typically Bayes factors between 1/100 and 100 are observed in medical research, and the reporting guidelines for JASP are therefore built on this scale [29]. While there are multiple scales for translating a Bayes factor into a qualitative statement about the evidence it resembles [27, 29, 30], these proposals do not differ drastically. One benefit is that by reporting the actual Bayes factor instead of “moderate evidence” or “strong evidence” researchers can quantify the evidence based on the reported Bayes factor themselves if desired. The oldest classification or labeling scheme goes back to Jeffreys [27], and the reporting guidelines of JASP are an adoption of the original Jeffreys scale. The JASP guidelines seperate between “anecdotal”, “moderate”, “strong”, “very strong” and “extreme” relative evidence for a hypothesis based on the size of the Bayes factor obtained.

**Fig. 1:**
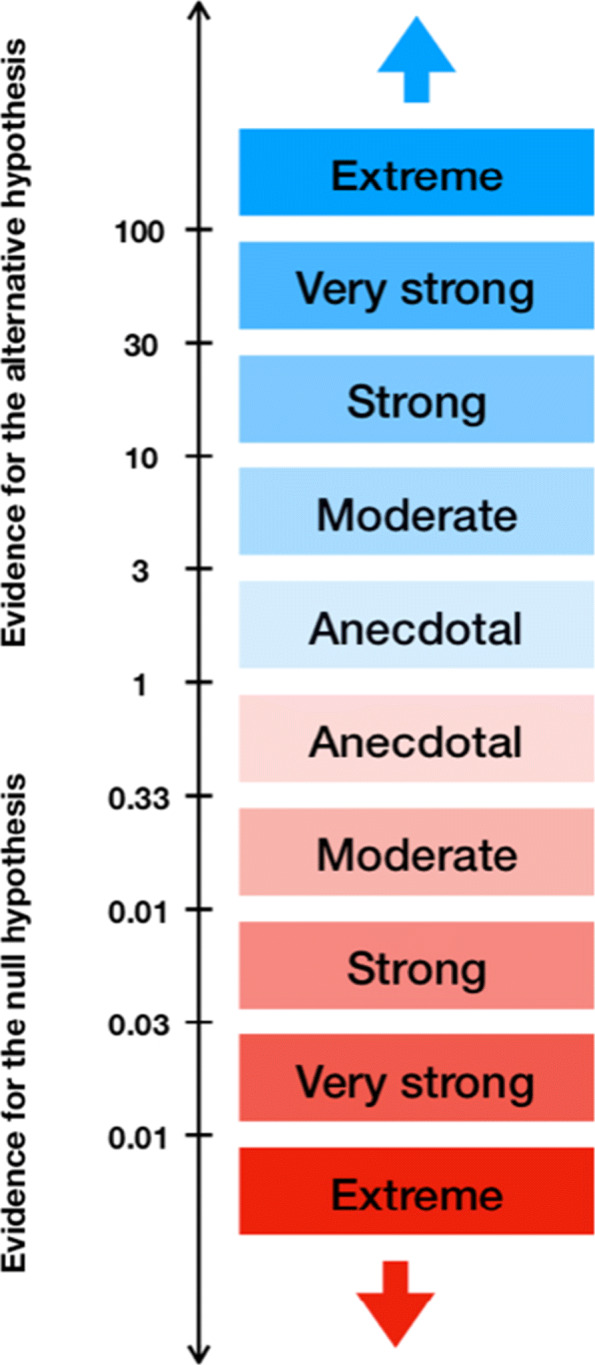
JASP classification scheme for the Bayes factor *BF*_10_

Figure 1 shows the classification scheme proposed for reporting results obtained in JASP. While the scale chosen is arbitrary, the scheme offers a good starting point for judging the relative evidence for the alternative hypothesis compared to the null hypothesis in light of the observed data *x*. Note that not all circumstances and research contexts require the same scaling: The obtained Bayes factor depends on the prior selected, so that heavily unrealistic hypothesis should require much larger Bayes factors to confirm the a priori unprobable statement in contrast to highly likely hypotheses, which have been confirmed in multiple previous studies already. A research hypothesis with low prior probability will therefore require a convincing Bayes factor such that the evidence overcomes the initial skepticism and the model attains considerable posterior credibility. Therefore, it is important to consider the prior odds carefully when performing such analyses instead of using isolated Bayes factors only. Nevertheless, the scheme provides a consensus which researchers can use for orientation when reporting results. In particular, it is a good starting point when a weakly informative prior is used. Such priors are prebuilt into JASP and can be selected there.

A more severe problem than their dependence on the prior with Bayes factors is that no matter what scale is used, they only state *relative* evidence instead of absolute evidence. This means that even a *BF*_10_=100 which states extreme evidence for the alternative over the null hypothesis only indicates that a change in beliefs about the hypotheses under consideration is necessitated strongly. But even then both hypotheses can be bad descriptions of the real underlying situation. Therefore, it is recommended always to report the labels with the prefix *relative*, that is in the above case one can state extreme evidence for *H*_1_ relative to *H*_0_, but not for *H*_1_ relative to any other set of or even possibly the set of all other hypotheses.

When the prior modelling is considered and it is kept in mind that the BF only states relative evidence for a hypothesis, the BF can safely be used to gauge the relative evidence for a hypothesis.

## Implementation

JASP is written in C++, using the Qt toolkit [18]. The analyses themselves are written in either R or C++ to improve the speed of especially simulation based methods. The display layer where the data in form of tables are rendered is written in javascript, and is built on top of jQuery UI and webkit.

Regarding the future, JASP is currently supported by some long-term grants that fund the JASP team of software developers, academics and students. The team includes four main software developers as well as several core members which have tenured positions. Of particular importance is the psychological methods group at the University of Amsterdam, which is dedicated to long-term support for JASP [31].

## Documentation and manual

Documentation of the implemented methods can be found at the official JASP site. There are both written tutorials as well as video tutorials which show how to conduct a given method. Also, the JASP reporting guidelines [29] offer an overview about some of the most important tests and methods available and how to report the results of an analysis. The official JASP site offers both a textbook for students [32] and additionally there is a textbook for learning statistics with JASP [33]. Both of these are free. Also, there are additional teaching materials and a user forum to support exchange and development of new features.

A particular nice feature of JASP is also given by the fact that it comes with an included data library, consisting of over 50 data sets to illustrate a variety of analyses.

In summary, documentation is rich and provides easy access and a flat learning curve.

## Flexibility and ease-of-use

JASP includes both frequentist and Bayesian methods, and this is a particular strength, as few competitors include that broad a palette of Bayesian methods. Next to this flexibility, ease-of-use is supported through an interactive live view where analyses are done in real time and added to the results page. The interface of JASP is intuitive and consists of a data page displaying the loaded data set, an analysis page, displaying the analyses which are carried out on this data set, and a results page which includes all results and plots of conducted analyses. In summary therefore, JASP can be judged as flexible and easy to use.

## Results

To study the behaviour of Bayesian methods in JASP, three typical questions arising in medical research are used as a scaffold: (1) Do multiple groups (treatment one, treatment two, control) differ on an observed metric variable, and if so, how large is the effect size? (2) Do two groups (treatment, control) differ on an observed metric variable, and if so, how large is the effect size between both groups? (3) How strong is the relationship between two observed variables? Usually NHST in form of (1) an analysis of variance (ANOVA) (2) a two-sample t-test and (3) linear regression is used to reject a null hypothesis via the use of *p*-values. In the following, it will be shown that Bayesian versions of these statistical procedures can complement NHST and provide even richer information for medical research. A compelling feature here is, that both traditional as well as the Bayesian methods can be run in JASP seamlessly [31, 34], so that methodological flexibility is guaranteed.

The aim of this paper therefore is to demonstrate JASPs ability to conduct Bayesian hypothesis testing and parameter estimation as well as NHST via p-values. However, it is argued that richer information is provided when shifting to the Bayesian paradigm, which allows for better medical decision making as currently often done in form of frequentist rejection of null hypotheses. Also, the results show that the transition can be achieved almost effortlessly, as JASP offers an intuitive graphical interface and covers a wide range of Bayesian counterparts for commonly used tests in medical research with rich annotations for correct interpretation and reporting.

Three datasets from medical research were used to compare NHST and Bayesian tests in JASP. The first dataset is from Moore and colleagues [35], and consists of 800 patients which had to exercise for six minutes. After the six minutes, heart rates of male and female patients were recorded. All patients were additionally classified as runners or sedentary patients, depending on averaging more than 15 miles per week or not, so that in total two treatment and two control groups of size 200 each sum up to 800 participants.

### Question (1) – analysis of variance (ANOVA)

A typical question in medical research would be to find out any differences between gender as well as both groups, leading to the setting of a 2×2 between subjects ANOVA for the variables group and gender. More specifically, a test for the hypothesis of differing average heart rates between gender and control and treatment groups is desired. The results of the frequentist ANOVA conducted in JASP are shown in Table 1. The output shows that both gender and group are significant variables as well as the interaction term for gender and group. All quantities of the ANOVA calculations, sum of squares, degrees of freedom, mean square, F-statistic, *η*^2^ and the *p*-value are given. Also, the Vovk-Sellke Maximum Ratio (VS-MPR*) is given based on the *p*-value, which is the maximum possible odds in favor of *H*_1_ over *H*_0_.

**Table 1:** ANOVA - Heart Rate

Cases	Sum of Squares	df	Mean Square	F	p	VS-MPR*	*η*^2^
Gender	45030.005	1.000	45030.005	185.980	<.001	1.296e+35	0.110
Group	168432.080	1.000	168432.080	695.647	<.001	1.264e+107	0.413
Gender * Group	1794.005	1.000	1794.005	7.409	0.007	11.062	0.004
Residual	192729.830	796.000	242.123				

Type III Sum of Squares

**Fig. 2:**
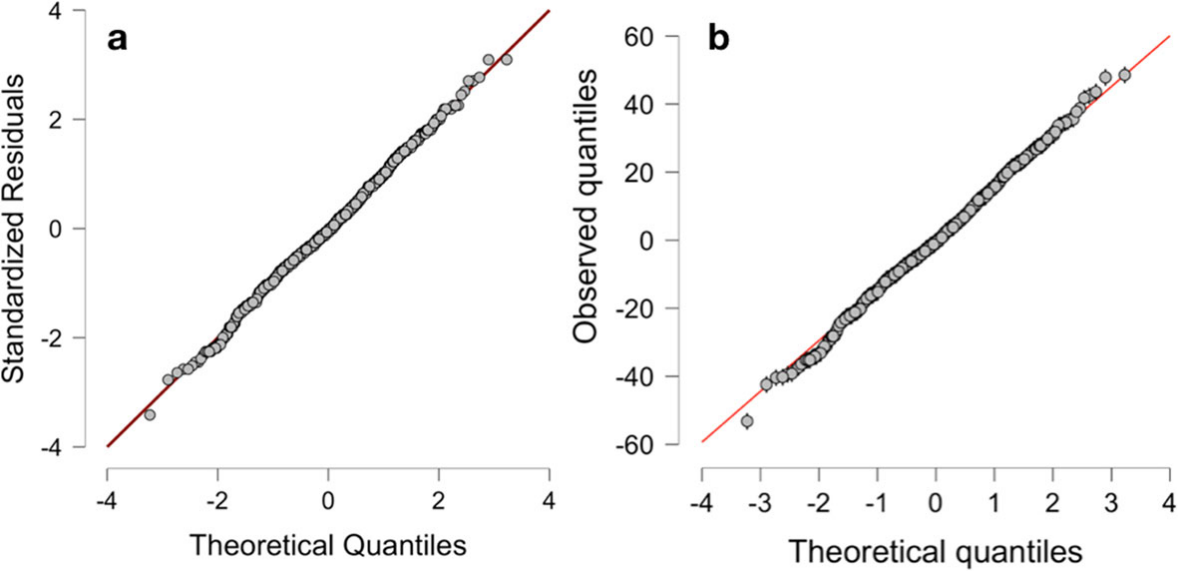
Q-Q-plots for the traditional and Bayesian ANOVA for the heart rate dataset of Moore and colleagues produced by JASP

One nice feature of JASP is that it offers the option to include assumption checks for the tests conducted: For the ANOVA, homogeneity of variance is required, and the included assumption check in form of Levene’s test is given in Table 2, showing that the assumption is violated. Still, investigating the provided Q-Q-plot in JASP (see Fig. 2a) shows that due to the balanced design of 200 participants in each sample and a high power due to 800 participants in total, the ANOVA will be relatively robust to the violations. Conducting a Bayesian ANOVA on the same data in JASP yields the results given in Table 3. There are five distinct models for each of which the prior probability *P*(*M*), the posterior probability *P*(*M*|*data*), the change from prior odds to posterior odds *BF*_*M*_ for each model, and the Bayes factor *BF*_10_ for the relative evidence of the alternative hypothesis *H*_1_ compared to the null hypothesis *H*_0_ as well as the error in percent is given. This is necessary, because for some analyses the results are based on numerical algoritms such as Markov chain Monte Carlo (MCMC), which yields an error percentage (for more details on the computation see [29]). The error percentage thus is an estimate of the numerical error in the computation of the Bayes factor via Gaussian quadrature in the BayesFactor R package [36] JASP uses internally, and values below 20% are deemed acceptable [37]. If the error percentage is deemed too high, the number of samples can be increased to reduce the error percentage at the cost of longer computation time. Also, the *BF*_*M*_ column shows the change from prior odds to posterior odds for each model. For example, for the full model including both main effects as well as their interaction effect, the prior odds are 0.2/(1−0.2)=0.25, while the posterior odds are 0.790/(1−0.790)=3.761905, leading to a ratio of 3.761905/0.25=15.04762, as shown in the *BF*_*M*_ column. All models are compared to the null model here, where the null model includes no predictor variables at all, and the full model includes both variables gender and group as well as their interaction term. It is clear that the *BF*_10_ of 3.463*e*+125 is largest for this last most complex model, indicating extreme evidence for this model according to Fig. 1 and the reporting guidelines for JASP [29]. Also, the *BF*_10_ column contains the Bayes factor that quantifies evidence for this model relative to the null model with no variables included, therefore it is 1 for the null model row. While the *BF*_*M*_ column thus states that the most complex model is the most probable a posteriori (because the prior odds were identical for all models, so that *BF*_*M*_ is largest iff *P*(*M*|*data*) is largest), the *BF*_10_ column also shows that the most complex model predicts the data best. Therefore, the Bayes factor indicates extreme evidence for the full model. It may be of interest to obtain a Bayes factor $BF_{10}(\mathcal {M}_{\text {main effects vs. full}})$ for comparison of the full model including the interaction effect, and the model with both main effects. This is straightforward, as due to the transitivity of the Bayes factor, it is clear that

$$\begin{array}{*{20}l} &\frac{BF_{10}\left(\mathcal{M}_{\text{main effects}}\right)}{BF_{10}\left(\mathcal{M}_{\text{full}}\right)}=\frac{\frac{p\left(x|H_{1}^{\mathcal{M}_{\text{main effects}}}\right)}{p\left(x|H_{0}^{\mathcal{M}_{\text{null}}}\right)}}{\frac{p\left(x|H_{1}^{\mathcal{M}_{\text{full}}}\right)}{p\left(x|H_{0}^{\mathcal{M}_{\text{null}}}\right)}}\\ &=\frac{p\left(x|H_{1}^{\mathcal{M}_{\text{main effects}}}\right)}{p\left(x|H_{1}^{\mathcal{M}_{\text{full}}}\right)}=BF_{10}\left(\mathcal{M}_{\text{main effects vs. full}}\right) \end{array} $$

**Table 2:** Test for Equality of Variances (Levene’s)

F	df1	df2	p	VS-MPR*
5.562	3.000	796.000	<.001	59.104

**Table 3:** Model comparison

Models	P(M)	P(M |data)	BF _*M*_	BF_10_	error %
Null model	0.200	2.281e-126	9.124e-126	1.000	
Gender + Group + Gender * Group	0.200	0.790	15.047	3.463e+125	2.485
Gender + Group	0.200	0.210	1.063	9.207e+124	1.068
Group	0.200	6.651e-36	2.661e-35	2.916e+90	2.683e-95
Gender	0.200	1.797e-107	7.186e-107	7.876e+18	2.699e-23

because the denumerators $p\left (x|H_{0}^{\mathcal {M}_{\text {null}}}\right)$ cancel each other out, so that dividing the main effects model Bayes factor $BF_{10}\left (\mathcal {M}_{\text {main effects}}\right)=9.207e+124$ by the full models Bayes factor $BF_{10}\left (\mathcal {M}_{\text {full}}\right)=3.463e+125$ yields a Bayes factor $BF_{10}\left (\mathcal {M}_{\text {main effects vs. full}}\right)\approx 0.2658677$ for comparing the main effects model to the full model, which also indicates that the full model is to be preferred. This Bayes factor can also be calculated in JASP by selecting *compare to best model* instead of *compare to null model* in the user interface. Figure 2b shows a Q-Q-plot for the residuals of the Bayesian ANOVA, showing that it is quite robust to the deviations from normality.

A compelling feature of the Bayesian statistical philosophy now is that posterior credible intervals on all variables of interest are easily obtained. While often frequentist confidence intervals are interpreted as containing the true parameter *θ* with 95% probability, this is actually the correct interpretation of a Bayesian credible interval, after observing the data *x*. Table 4 shows the model averaged posterior summaries of the full model for both variables and the interaction term.

**Table 4:** Model averaged posterior summary

				95% Credible Interval	
Variable	Level	Mean	SD	Lower	Upper
Intercept		124.490	0.551	123.168	125.426
Gender	Female	7.448	0.559	6.339	8.553
	Male	-7.448	0.559	-8.586	-6.373
Group	Control	14.474	0.557	13.334	15.551
	Runners	-14.474	0.557	-15.584	-13.367
Gender * Group	Female & Control	1.465	0.547	0.378	2.577
	Female & Runners	-1.465	0.547	-2.586	-0.387
	Male & Control	-1.465	0.547	-2.586	-0.387
	Male & Runners	1.465	0.547	0.378	2.577

From the table, one can easily see that females have a posterior mean of 7.448, that is an increased heart rate of 7.448 beats per minute, while males have a posterior mean of −7.448, indicating a decreased heart rate of the same magnitude compared to the global mean. Thus, the heart rate seems to be differing between males and females. Specifically, for females with 95% probability after observing the data *x* the average heart beat lies in the range of values [6.339,8.553], so that with 95% we can be sure that females have an increased heart rate of at least 6.339≈6 beats per minute after exercising 6 minutes compared to the global mean. The 95% credible intervals of males and females do not overlap, so we can be quite confident that there is a true difference.

**Fig. 3:**
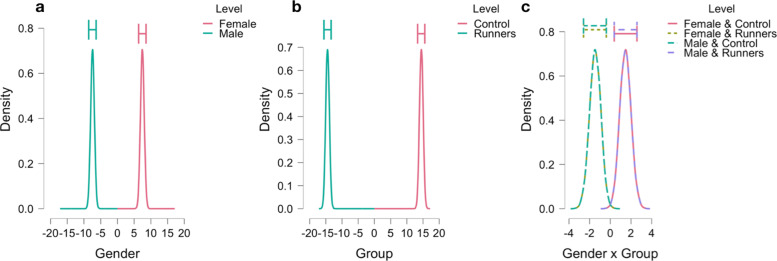
Posterior plots for all variables and interaction terms for the heart rate data of Moore and colleagues produced by JASP

Other inferences are obtained in identical manner from Table 4. Note that the frequentist MLE estimates and confidence intervals cannot offer this flexibility. The values in Table 4 can also be obtained as plots in JASP, showing the posterior densities, see Fig. 3a-c.

### Question (2) – paired samples t-test

Another common situation in medical research is the paired samples t-test which compares the means *μ*_1_ and *μ*_2_ of the same population at two different timepoints (pre-treatment vs. after treatment). The dataset used is again from Moore and colleagues [35], and provides the number of disruptive behaviours by dementia patients during two different phases of the lunar cycle. The hypothesis tested is *H*_0_: “Average number of disruptive behaviours in patients with dementia does not differ between full moon and other days” against the alternative *H*_1_ of a differing average numbers of disruptive behaviours. Table 5 shows the results of the frequentist paired-samples t-test, indicating with *p*<.001 that *H*_0_ can be rejected. The paired samples *t*-test therefore suggests that the data (or more extreme data) are unlikely to be observed if the average number of disruptive behaviours was identical during full moon days and other days in patients with dementia. Note that this is not what researchers actually want to know: The desired answer is which hypothesis is more probable after observing the data, which is exactly quantified by the posterior odds $\mathbb {P}(H_{1}|x)/\mathbb {P}(H_{0}|x)$, of which the *BF*_10_ is a key ingredient (remember that the posterior odds are the product of the Bayes factor and the prior odds). A large *BF*_10_ therefore necessitates a change in beliefs towards *H*_1_. Assumption checks include a Shapiro-Wilk test on normality, which is not significant with *p*=.148. Now, the Bayesian paired-samples t-test shown in Table 6 yields *BF*_10_=1521.058, indicating extreme evidence for *H*_1_. JASP produces also a plot of the prior and posterior distribution of the effect size *δ* according to Cohen [25], which is of interest in most medical research settings [29].

**Table 5:** Paired samples T-Test

		t	df	p	Mean Difference
Moon -	Other	6.452	14	<.001	2.433

**Table 6:** Bayesian Paired Samples T-Test

			BF_10_	error %
Moon	-	Other	1521.058	5.014e-7

**Fig. 4:**
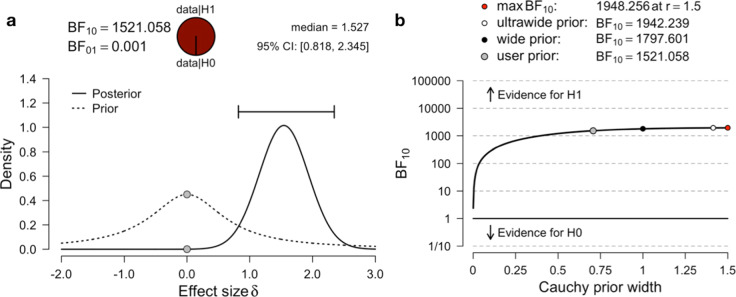
Prior and posterior plot and robustness check for the heart dementia data of Moore and colleagues produced by JASP

Figure 4a shows this prior and posterior plot of the effect size *δ* as well as the produced *BF*_10_. A large advantage of the Bayesian paradigm reveals itself here: The posterior of the effect size *δ* precisely estimates which effect size is most probable after observing the data *x*. The frequentist paired-samples t-test did not yield any information about the effect size. Although the test was significant, it did not state anything about whether the observed effect is small, medium or large. The prior-posterior plot shows how the prior probability mass is reallocated to the posterior via observing the data and shows that with 95% probability, the true effect size *δ* is in [0.818,2.345] and the posterior median is 1.527, indicating a large effect. Another benefit is given by the robustness check plot given in Fig. 4b: Different prior distribution widths are used for the effect size *δ* and the Bayes factor *BF*_10_ is computed. Specifically, the prior width of the Cauchy prior *C*(0,*γ*) on the effect size *δ* is increased gradually, showing how the prior shape influences the resulting *BF*_10_. Figure 4b shows that even when changing the prior from the user prior, which equals a medium $C(0,\sqrt {2}/2)$ prior, to a wide *C*(0,1) or even ultrawide $C(0,\sqrt {2})$ prior, the Bayes factor for *H*_1_ stays above 1000. Thus, the influence of the prior is negligible here, so that only an inconsequential amount of subjectivity goes into the analysis.

### Question (3) – linear regression

One of the most widespread methods in biomedical research and clinical trials is linear regression [4]. The dataset used here is from Mestek, Plaisance and Grandjean [38] published in the *Journal of American College Health*. The study provided 100 participants’ Body Mass Index (BMI) and average daily number of steps, investigating this relationship with linear regression models.

**Fig. 5:**
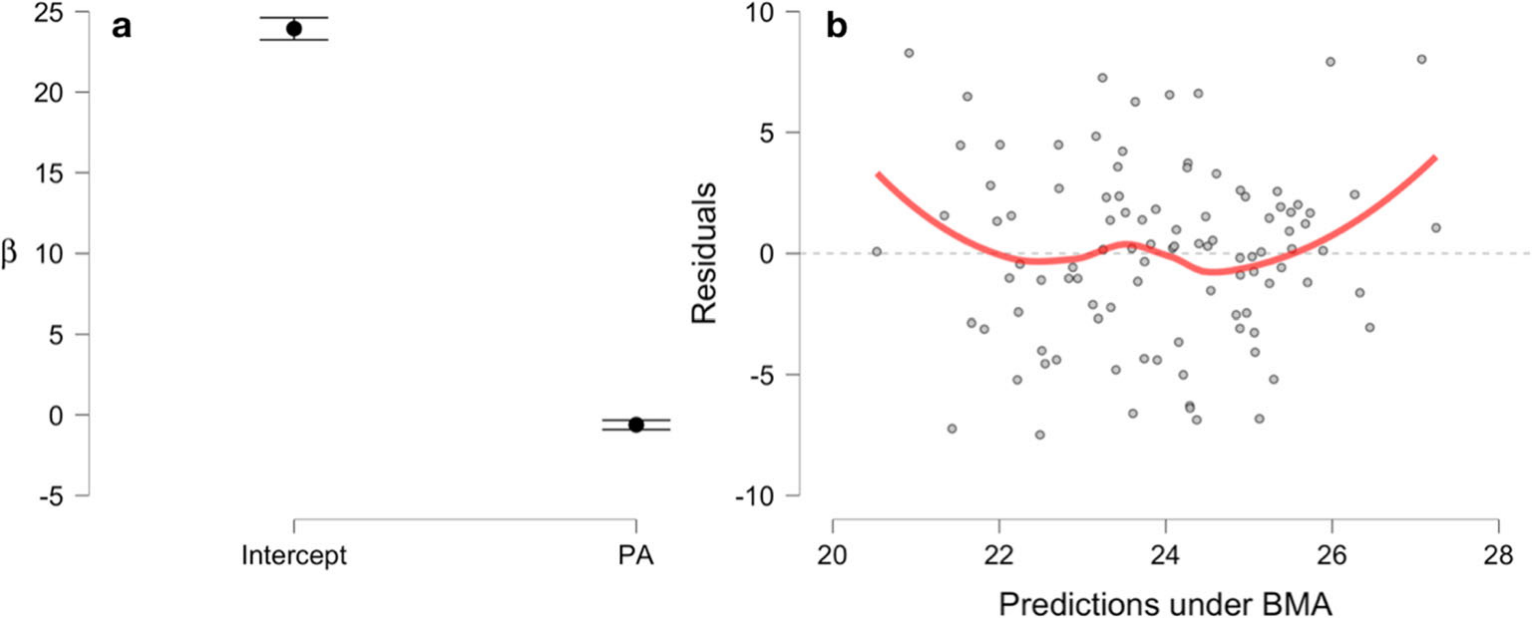
Posterior coefficients with credible intervals and residual plot for the BMI data of Mestek et al. produced by JASP

A traditional linear regression with the BMI as dependent variable and the average number of daily steps (in thousands) of participants as explanatory variable yields the results given in Table 7. The table shows that physical activity (PA) is a significant predictor of the BMI of participants, as *p*<.001. While JASP also offers to provide confidence intervals, these are counterintuitive to interpret, and therefore the Bayesian linear regression given in Table 8 is preferred. Again, the change from prior to posterior odds for the model *BF*_*M*_ and the Bayes factor for the alternative *BF*_10_ are given, as well as the models prior probability *P*(*M*) and the posterior model probability *P*(*M*|*data*) after observing the data. One can conclude from the results, that the *BF*_*M*_=284.327 of the physical activity model shows extreme evidence for the model including the variable. Also, the identical *BF*_10_ for the alternative *H*_1_ relative to *H*_0_, where *H*_1_ states that the regression coefficient for the PA variable differs from zero, shows that the coefficient for the variable is most probable non-zero. The null hypothesis *H*_0_ of a regression coefficient of size zero for the PA variable can thus be rejected based on this result, and even better, the alternative *H*_1_ can be regarded as *confirmed*, which would *not* be allowed when using *p*-values because accepting hypotheses is generally not allowed in frequentist NHST when interpreted in the sense of Ronald Fisher’s significance testing. Note that when interpreted from the Neyman-Pearson theory of hypothesis testing, accepting a hypothesis is allowed, but as the Neyman-Pearson theory is only concerned with long-term type I error control, nothing can be said about the hypothesis tested in the performed study or experiment. As Neyman and Pearson (see [39], p. 291) state explicitly, their theory *“tells us nothing as to whether in a particular case H is true”*. Also, the PA model explains 15% of the variance observed in the data as can be seen from Table 8. Again in this situation, Table 9 shows the posterior summary of coefficients for the Bayesian linear regression, yielding 95% credible intervals so that inference about the most probable range of coefficient values given the data *x* can be made. Figure 5a shows a plot of the posterior coefficients obtained from the Bayesian linear regression for the BMI data produced by JASP. The Mean and 95% credible intervals are shown, indicating that the PA coefficient is with 95% probability smaller than −0.326, compare Table 9. Figure 5b shows a residual plot to check the assumption of normally distributed residuals, which seems fine for the Bayesian linear regression model. Note that JASP internally uses the BAS package for R [40] for the computations.

**Table 7:** Frequentist linear regression for the BMI data set

	Unstandardized	Std. Error	t	p
(Intercept)	29.578	1.412	20.948	<.001
PA	-0.655	0.158	-4.135	<.001

**Table 8:** Bayesian linear regression for the BMI data set

Models	P(M)	P(M |data)	BF_M_	BF_10_	R^2^
Null model	0.500	0.004	0.004	1.00	0.00
PA	0.500	0.996	284.327	284.33	0.15

**Table 9:** Posterior summaries of coefficients

						95% Credible Interval	
Coefficient	Mean	SD	P(incl)	P(incl|data)	BF_*inclusion*_	Lower	Upper
Intercept	23.939	0.366	1.000	1.000	1.000	23.244	24.615
PA	-0.609	0.157	0.500	0.996	284.327	-0.908	-0.326

## Discussion

The comparison of NHST and Bayesian methods conducted reveals that the Bayesian approach complements the traditional frequentist tests and provides even richer information for hypothesis testing and parameter estimation. Also, both of these benefits can be achieved with JASP easily.

Not only can Bayes factors be used to quantify the relative evidence for the alternative hypothesis *H*_1_ compared to *H*_0_ in JASP, but additional parameter estimation with easy to interpret credible intervals makes inference more seamless compared to traditional methods. Also, model comparisons and robustness checks can be included into the main analysis to assess the degree to which the conclusions change with background assumptions like the chosen priors, no matter if a t-test, an analysis of variance or a linear regression model is the method of choice.

Also, detailed plots and visualisations of results are obtained quickly, allowing easier interpretation and communication of analysis results. What is more, a complete analysis in JASP can be saved in a single JASP-file, making it possible to send a conducted analysis to a colleague or even share it publicly. This fosters reproducibility and makes checking results easier for colleagues and reviewers of journals. In contrast, SPSS, Stata or R are less transparent as they often depend on the used libraries and version or require detailed programming knowledge, making reanalysing an original dataset much more complicated and time-consuming.

Bayesian inference in JASP also profits from credible intervals and posterior estimates which are more interpretable than traditional MLE estimates with confidence intervals, and allows for a unified judgement of evidence for a model or hypothesis in form of the Bayes factor. Note that there is a large palette of more options for each method (like prior specification, descriptive statistics, providing *BF*_01_ instead of *BF*_10_, inclusion probability for coefficients, and so on) not described here due to space reasons. Thus, JASP provides many desirable features for the methods implemented, making it a full-grown alternative to statistics packages like SPSS or Stata while also providing an equally intuitive user interface. A definite advantage of JASP is its ability to conduct a multitude of Bayesian tests in comparison to SPSS or Stata, as well as being free for everyone.

Still, although a good spectrum of statistical tests and methods is available in JASP, there are also limitations. Especially for medical research there are some important methods missing. For example, JASP offers no options for survival analysis, which is essential in clinical trials [41, 42]. Also, more complex generalized linear models are missing, for example there is no Bayesian logistic regression available, a method of large importance for medical research [43]. On the other hand, recently, machine learning algorithms like clustering, penalized regression models, linear discriminant analysis and classification and regression trees have been added in form of a machine learning module.

## Conclusion

To review JASP, three worked out examples of common situations in biomedical research were provided in this paper, consisting of an ANOVA, a paired t-test and a linear regression model. Conducting and interpreting an analysis in JASP is straigthforward and guided by an intuitive interface with lots of buttons for explanations, while assumptions of a wide variety of tests can be included into the main analysis via a single mouse click. This is a large benefit to competitors like SPSS or Stata, as these do not offer such a wide range of Bayesian methods and are more complicated, having a steeper learning curve and long manuals.

The program interface, documentation and manuals are intuitive and allow the user to quickly accommodate to JASP. The flexibility gained by including NHST and Bayesian methods is a key advantage of JASP compared to other software, and the performance is flawless as shown by the worked out examples.

In summary, the results show that JASP provides easy access to advanced (Bayesian) statistical methods, and NHST is easily complemented by Bayesian methods. Also, the effect size, often of large relevance in medical research, can be easily estimated in JASP via Bayesian methods for a variety of tests, and this offers another advantage compared to frequentist methods.

In summary, in its current state JASP offers a wide range of suitable tests routinely used in medical research and allows seamless transition from NHST to Bayesian inference. This shift towards Bayesian alternatives for null hypothesis significance testing could substantially improve the reproducibility and validity of biomedical research in science.

## Availability and requirements

Project name: JASP Project home page: https://doi.org/https://jasp-stats.org/Operating system(s): e.g. Platform independent Programming language: C++, R Other requirements: NoneLicense: Free and open source (FOSS) Any restrictions to use by non-academics: None

## Supplementary information

**Additional file 1** PDF export of JASP-file for the heart rate data analysis

**Additional file 2** PDF export of JASP-file for the dementia patient data analysis

**Additional file 3** PDF export of JASP-file for the physical activity and BMI data analysis

## Data Availability

All datasets analysed are available in the JASP standard installation as demonstration data sets, so these can easily be obtained via installing JASP. All results and analyses have been appended as Supplementary files, too.

## Abbreviations

BF: Bayes factor
NHST: Null hypothesis significance testing
JASP: Jeffreys’awesome statistics package (software)
ANOVA: Analysis of variance
SPSS: Statistics package for the social sciences (software)
PA: Physical activity

## References

[1] Altman DG (1982). Statistics in medical journals. Stat Med.

[2] Altman DG, Gore SM, Gardner MJ, Pocock SJ (1983). Statistical guidelines for contributors to medical journals. Br Med J (Clin Res ed.).

[3] Altman DG (1991). Statistics in medical journals: Developments in the 1980s. Stat Med.

[4] Altman DG (1991). Practical Statistics for Medical Research.

[5] Fisher RA (1925). Statistical Methods for Research Workers.

[6] Neyman J, Pearson ES (1936). Contributions to the theory of testing statistical hypotheses. Stat Res Mem.

[7] Colquhoun D (2014). An investigation of the false discovery rate and the misinterpretation of *p*-values. R Soc Open Sci.

[8] Benjamin DJ, Berger JO (2019). Three recommendations for improving the use of *p*-values. The Am Stat.

[9] Ioannidis JPA (2005). Why most published research findings are false. PLoS Med.

[10] Wasserstein RL, Lazar NA (2016). The ASA’s statement on *p*-values: context, process, and purpose. The Am Stat.

[11] Wasserstein RL, Schirm AL, Lazar NA (2019). Moving to a world beyond “*p*<0.05”. The Am Stat.

[12] Matthews R, Wasserstein R, Spiegelhalter D (2017). The ASA’s *p*-value statement, one year on. Significance.

[13] Colquhoun D. The problem with *p*-values. 2016. https://aeon.co/essays/it-s-time-for-science-to-abandonthe-term-statistically-significant. Accessed 11 Oct 2016.

[14] Ioannidis JPA (2019). What have we (not) learnt from millions of scientific papers with *p*-values?. The Am Stat.

[15] Colquhoun D (2017). The reproducibility of research and the misinterpretation of *p*-values. R Soc Open Sci.

[16] Ioannidis JPA (2016). Why most clinical research is not useful. PLoS Med.

[17] McElreath R, Smaldino PE (2015). Replication, communication, and the population dynamics of scientific discovery. PLoS ONE.

[18] JASP Team. JASP (Version 0.12)[Computer software]. 2020. https://jasp-stats.org/.

[19] Jeffreys H (1931). Scientific Inference.

[20] R Core, Team. R: A language and environment for statistical computing. R Found Stat Comput. 2019. https://www.r-project.org/.

[21] Open Science Foundation. OSF - Open Science Foundation. https://osf.io/. Accessed 25 Oct 2019.

[22] McElreath R (2016). Statistical Rethinking: A Bayesian Course With Examples in R and Stan.

[23] Robert C, Casella G (2004). Monte Carlo Statistical Methods.

[24] Kruschke JK (2015). Doing Bayesian Data Analysis: A Tutorial with R, JAGS, and Stan, Second Edition.

[25] Cohen J (1988). Statistical Power Analysis for the Behavioral Sciences.

[26] Rouder JN, Speckman PL, Sun D, Morey RD, Iverson G (2009). Bayesian t tests for accepting and rejecting the null hypothesis. Psychon Bull Rev.

[27] Jeffreys H (1961). Theory of Probability.

[28] Gelman A, Lee D, Guo J (2015). Stan: A probabilistic programming language for Bayesian inference. J Educ Behav Stat.

[29] van Doorn J, van den Bergh D, Bohm U, Dablander F, Derks K, Draws T, Evans NJ, Gronau QF, Hinne M, Kucharský Š, Ly A, Marsman M, Matzke D, Raj A, Sarafoglou A, Stefan A, Voelkel JG, Wagenmakers E-J. The JASP guidelines for conducting and reporting a Bayesian analysis. PsyArxiv Preprint. 2019. doi:10.31234/osf.io/yqxfr. 10.3758/s13423-020-01798-5 PMC8219590 33037582

[30] Good IJ (1950). Probability and the Weighing of Evidence.

[31] Wagenmakers EJ, Love J, Marsman M, Jamil T, Ly A, Verhagen J, Selker R, Gronau QF, Dropmann D, Boutin B, Meerhoff F, Knight P, Raj A, van Kesteren EJ, van Doorn J, Šmíra M, Epskamp S, Etz A, Matzke D, de Jong T, van den Bergh D, Sarafoglou A, Steingroever H, Derks K, Rouder JN, Morey RD (2018). Bayesian inference for psychology. Part II: Example applications with JASP. Psychon Bull Rev.

[32] Goss-Sampson MA. Statistical analysis in JASP 0.10.2: A guide for students; 2019.

[33] Navarro DJ, Foxcroft DR, Faulkenberry TJ. Learning statistics with JASP: A tutorial for psychology students and other beginners; 2019. https://learnstatswithjasp.com/.

[34] Etz A, Vandekerckhove J (2016). A Bayesian perspective on the reproducibility project: Psychology. PLoS ONE.

[35] Moore DS, McCabe GP, Craig BA (2012). Introduction to the Practice of Statistics.

[36] Morey RD, Rouder JN. BayesFactor: Computation of Bayes factors for common designs. 2018. https://cran.r-project.org/package=BayesFactor.

[37] van den Bergh D, van Doorn J, Marsman M, Draws T, van Kesteren E, Derks K, Wagenmakers E. A Tutorial on Conducting and Interpreting a Bayesian ANOVA in JASP. 2019. 10.31234/osf.io/spreb.

[38] Mestek ML, Plaisance E, Grandjean P (2008). The relationship between pedometer-determined and self-reported physical activity and body composition variables in college-aged men and women. J Am Coll Health.

[39] Neyman J, Pearson ES (1933). On the problem of the most efficient tests of statistical hypotheses. Phil Trans R Soc Lond. A.

[40] Clyde M. Bayesian variable selection and model averaging using Bayesian adaptive sampling. R Package Version 1.5.5.R Package Version 1.5.5. 2018.

[41] Klein JP, van Houwelingen HC, Ibrahim JG, Scheike TH (2014). Handbook of survival analysis.

[42] Ibrahim JG, Chen M-H, Sinha D (2001). Bayesian Survival Analysis.

[43] Faraway JJ (2016). Extending the Linear Model with R : Generalized Linear, Mixed Effects and Nonparametric Regression Models.

