# Supplementary material for: Bayesian alternatives to null hypothesis significance testing in biomedical research: a non-technical introduction to Bayesian inference with JASP
Source: BMC Med Res Methodol. 2020 Jun 5;20:142. doi: 10.1186/s12874-020-00980-6 (PMC7275319; doi:10.1186/s12874-020-00980-6)

Heart Rate: Factorial 2 x 2 Between Subjects ANOVA

Description:

This data set, "Heart Rate", provides heart rates of male and female runners and generally sedentary participants following 6 minutes exercise.

Variables:

- **Gender** - Participant's gender (Female, Male).
- **Group -** Group of 'Runners' (averaging more than15 miles per week) and 'Control' group (generally sedentary participant).
- **Heart Rate -** Heart rate after six minutes of exercise.

This example JASP file demonstrates the use of a 2 x 2 between subjects ANOVA. Specifically, we test whether heart rates differ between gender and groups.

References:

Moore, D. S., McCabe, G. P., and Craig, B. A. (2012). *Introduction to the Practice of Statistics* (7th ed.). New York: Freeman.

Wood, P.D, Haskell, W. L., Stern, M. P., Lewis, S. and Perry, C. (1977). Plasma lipoprotein distributions in male and female runners. *Annals of the New York Academy of Sciences,* 301: 748-763.

ANOVA

We conduct a two-way ANOVA of differences between Males and Females, Runners and Controls and their interaction. All terms are statistically significant. Note that the interaction explains only 0.4% of the variance of heart rates.

ANOVA - Heart Rate

Cases	Sum of Squares	df	Mean Square	F	p	VS-MPR*	η^2
Gender	45030.005	1.000	45030.005	185.980	< .001	1.296e +35	0.110
Group	168432.080	1.000	168432.080	695.647	< .001	1.264e +107	0.413
Gender ∗ Group	1794.005	1.000	1794.005	7.409	0.007	11.062	0.004
Residual	192729.830	796.000	242.123				

Note. Type III Sum of Squares

* Vovk-Sellke Maximum *p* -Ratio: Based on the *p* -value, the maximum possible odds in favor of H_1 over H_0 equals 1/(-e *p* log(*p*)) for $p \leq .37$ (Sellke, Bayarri, & Berger, 2001).

Assumption Checks

Test for Equality of Variances (Levene's)

F	df1	df2	p	VS-MPR*
5.562	3.000	796.000	< .001	59.104

* Vovk-Sellke Maximum *p* -Ratio: Based on the *p* -value, the maximum possible odds in favor of H_1 over H_0 equals 1/(-e *p* log(*p*)) for $p \leq .37$ (Sellke, Bayarri, & Berger, 2001).

Levene's test of equal variances suggests that the assumption of normality is violated. However, the balanced design (200 participants in each sample) and high power (800 participants in total) mean that the ANOVA will be relatively robust (see descriptives at the bottom). See also Richter & Peyton (2003).

Q-Q Plot

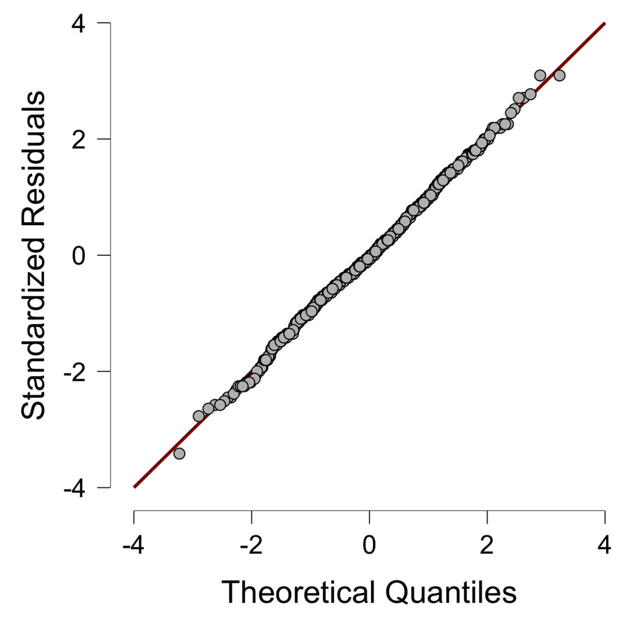

The Q-Q plot suggests no violation of normality.

Marginal Means

Marginal Means - Gender

Gender	Marginal Mean	SE	95% CI Lower	95% CI Upper
Female	131.993	0.778	130.465	133.520
Male	116.987	0.778	115.460	118.515

Marginal Means - Group

Group	Marginal Mean	SE	95% CI Lower	95% CI Upper
Control	139.000	0.778	137.473	140.527
Runners	109.980	0.778	108.453	111.507

Descriptives

Descriptives - Heart Rate

Gender	Group	Mean	SD	N
Female	Control	148.000	16.271	200
	Runners	115.985	15.972	200
Male	Control	130.000	17.100	200
	Runners	103.975	12.499	200

Descriptives Plot

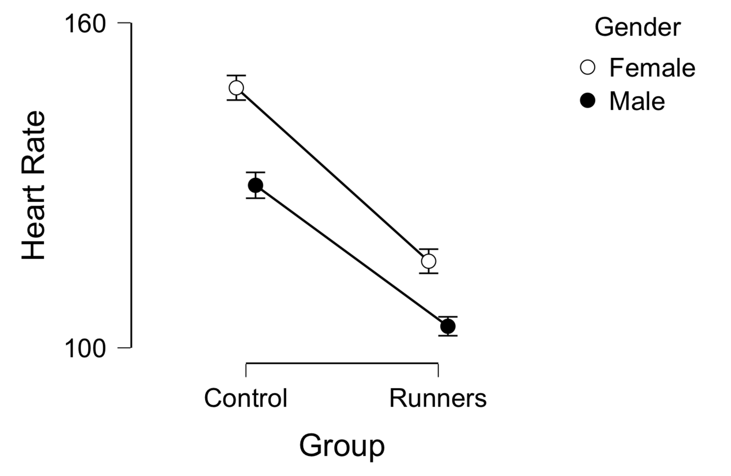

The above plot shows the results. Error bars depict 95% CIs. There is a clear difference between the Control group and the group of Runners, both for Females and Males. Women appear to have a higher heart rate than men. The fact that the two lines are not exactly parallel signifies the interaction effect, which suggests that the differences between the Males and Females is larger in the Control group than in the group of Runners.

Descriptive Statistics

Descriptive Statistics

	Heart Rate Control	Heart Rate Runners
Valid	400	400
Missing	0	0
Mean	139.000	109.980
Variance	359.088	241.298

Descriptive Statistics

Descriptive Statistics

	Heart Rate Female	Heart Rate Male
Valid	400	400
Missing	0	0
Mean	131.993	116.987
Variance	516.148	393.516

Bayesian ANOVA

Model Comparison

Models	P(M)	P(M|data)	BF_M	BF_{10}	error %
Null model	0.200	2.281e -126	9.124e -126	1.000	
Gender + Group + Gender ∗ Group	0.200	0.790	15.047	3.463e +125	2.485
Gender + Group	0.200	0.210	1.063	9.207e +124	1.068
Group	0.200	6.651e -36	2.661e -35	2.916e +90	2.683e -95
Gender	0.200	1.797e -107	7.186e -107	7.876e +18	2.699e -23

Analysis of Effects - Heart Rate

Effects	P(incl)	P(incl|data)	BF_{incl}
Gender	0.600	1.000	∞
Group	0.600	1.000	∞
Gender ∗ Group	0.200	0.790	15.047

Model Averaged Posterior Summary

Variable	Level	Mean	SD	95% Credible Interval Lower	95% Credible Interval Upper
Intercept		124.490	0.551	123.168	125.426
Gender	Female	7.448	0.559	6.339	8.553
	Male	-7.448	0.559	-8.586	-6.373
Group	Control	14.474	0.557	13.334	15.551
	Runners	-14.474	0.557	-15.584	-13.367
Gender ∗ Group	Female & Control	1.465	0.547	0.378	2.577
	Female & Runners	-1.465	0.547	-2.586	-0.387
	Male & Control	-1.465	0.547	-2.586	-0.387
	Male & Runners	1.465	0.547	0.378	2.577

Model Averaged Posterior Distributions

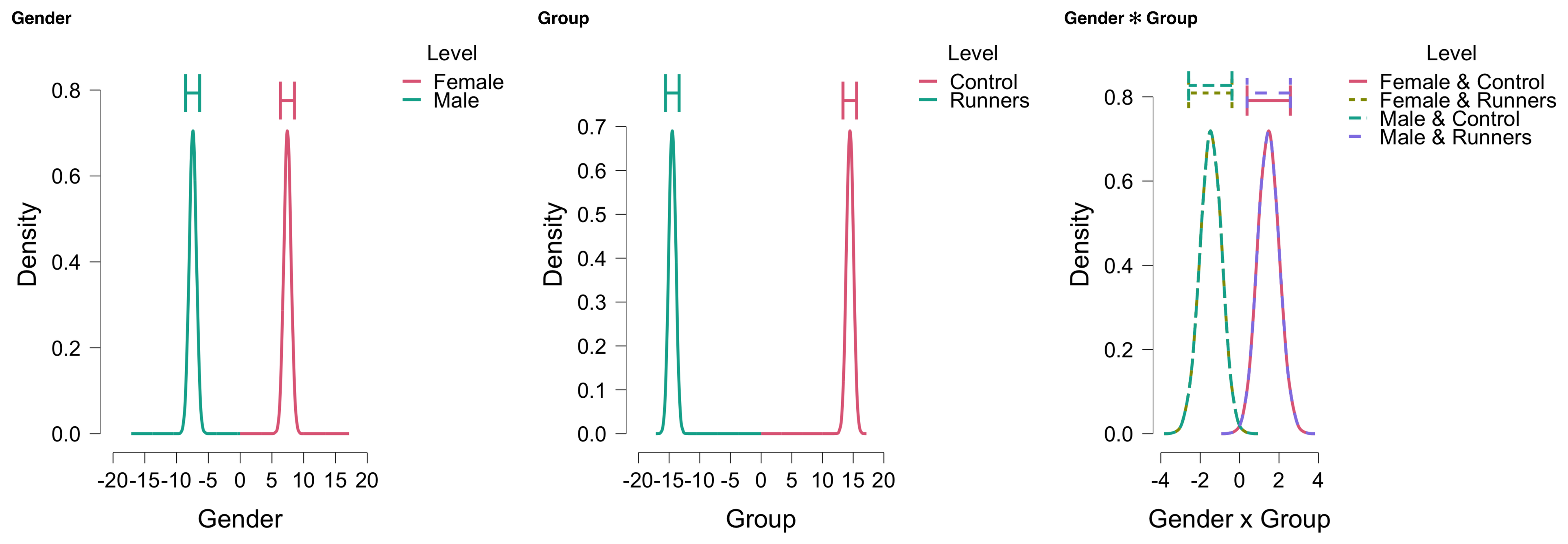

Model Averaged Q-Q Plot

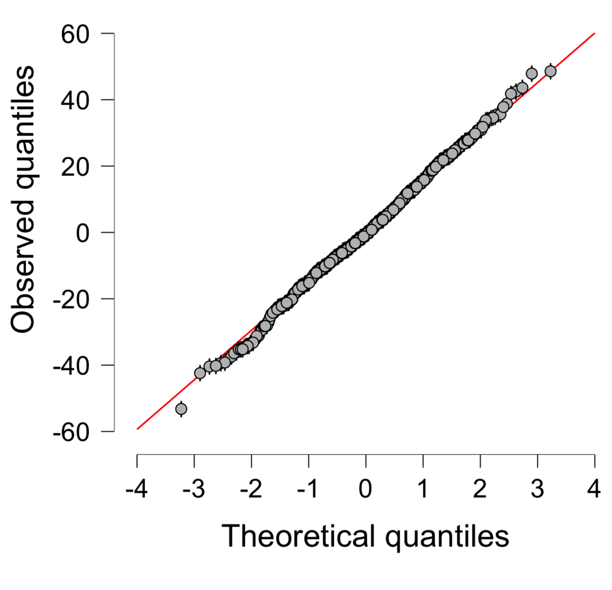

Supplement: Supplementary file 1 — Additional file 1 PDF export of JASP-file for the heart rate data analysis [file 12874_2020_980_MOESM1_ESM.pdf]
