# Supplementary material for: Bayesian alternatives to null hypothesis significance testing in biomedical research: a non-technical introduction to Bayesian inference with JASP
Source: BMC Med Res Methodol. 2020 Jun 5;20:142. doi: 10.1186/s12874-020-00980-6 (PMC7275319; doi:10.1186/s12874-020-00980-6)

Moon and Aggression: Paired Samples T-Test

Description:

This data set, "Moon & Aggression", provides the number of disruptive behaviors by dementia patients during two different phases of the lunar cycle (Moore et al, 2012, p. 410). Each row corresponds to one participant.

Variables:

- **Moon** - The average number of disruptive behaviors during full moon days.
- **Other** - The average number of disruptive behaviors during other days.

This example JASP file demonstrates the use of paired samples *t*-test. Specifically, we will examine the adequacy of the null hypothesis which states that the average number of disruptive behaviors among patients with dementia does not differ between moon days and other days.

References:

Moore, D. S., McCabe, G. P., and Craig. B. A. (2012) *Introduction to the Practice of Statistics* (7th ed). New York: Freeman.

"These data were collected as part of a larger study of dementia patients conducted by Nancy Edwards and Alan Beck, Purdue University." (Moore et al, 2012, p. N-8).

Paired Samples T-Test

Paired Samples T-Test

			t	df	p	Mean Difference	SE Difference
Moon	-	Other	6.452	14	< .001	2.433	0.377

Note. Student's t-test.

The paired samples *t*-test suggests that the data (or more extreme) are unlikely to occur if the average number of disruptive behaviors was the same during full moon days and other days.

Assumption Checks

The assumption of normality of differences is not significant. We provisionally retain the null hypothesis that the data are normally distributed.

Test of Normality (Shapiro-Wilk)

			W	p
Moon	-	Other	0.913	0.148

Note. Significant results suggest a deviation from normality.

Descriptives

During full moon days, patients show disruptive behavior more frequently than during other days.

Descriptives

	N	Mean	SD	SE
Moon	15	3.022	1.499	0.387
Other	15	0.589	0.445	0.115

Descriptives Plot

Moon - Other

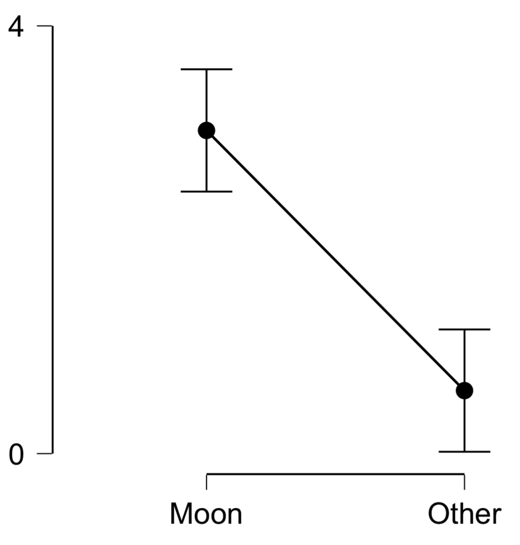

Bayesian Paired Samples T-Test

Bayesian Paired Samples T-Test

			BF_{10}	error %
Moon	-	Other	1521.058	5.014e-7

Descriptives

Descriptives Plot

Moon - Other

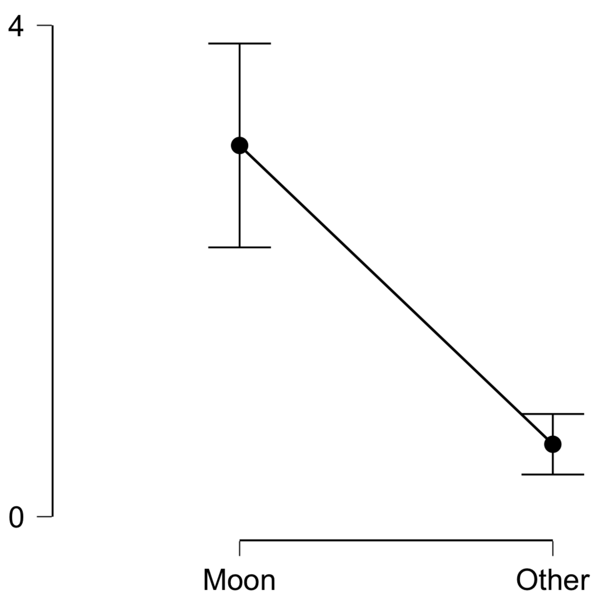

Inferential Plots

Moon - Other

Prior and Posterior

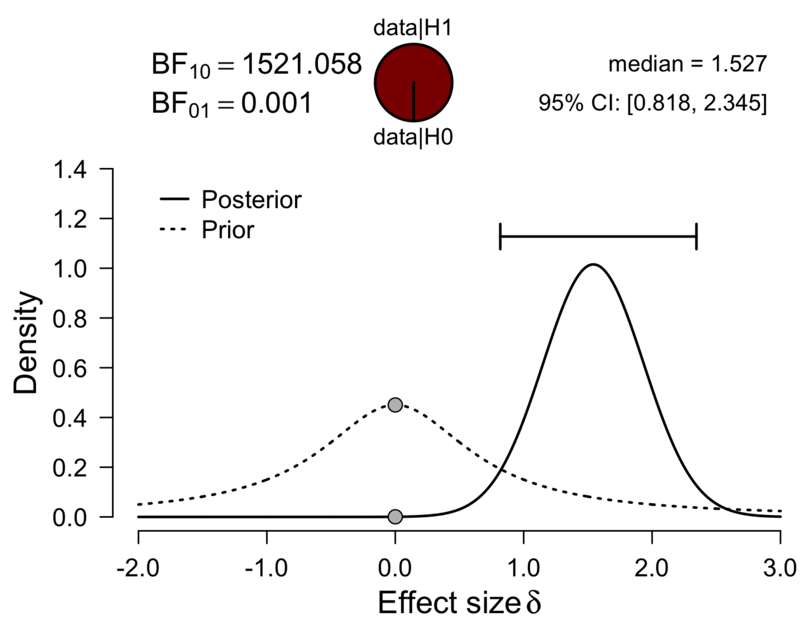

Bayes Factor Robustness Check

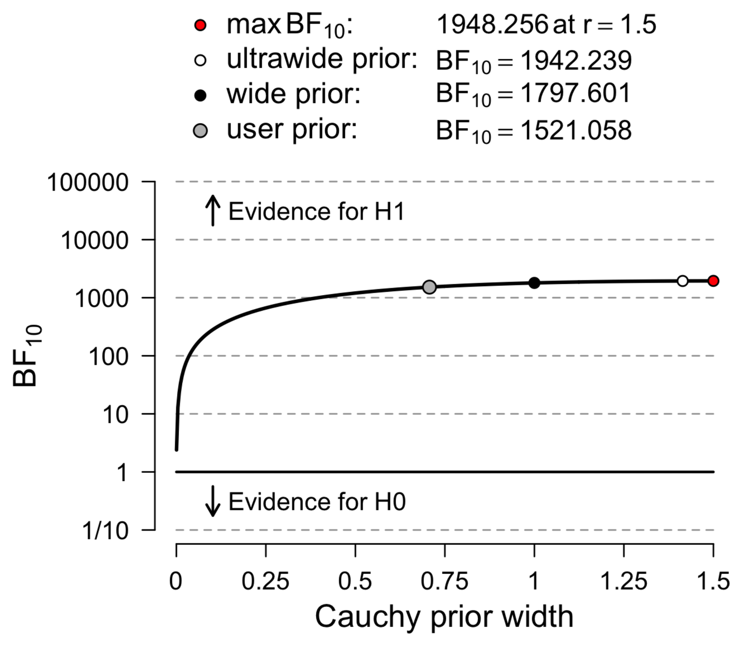

Sequential Analysis

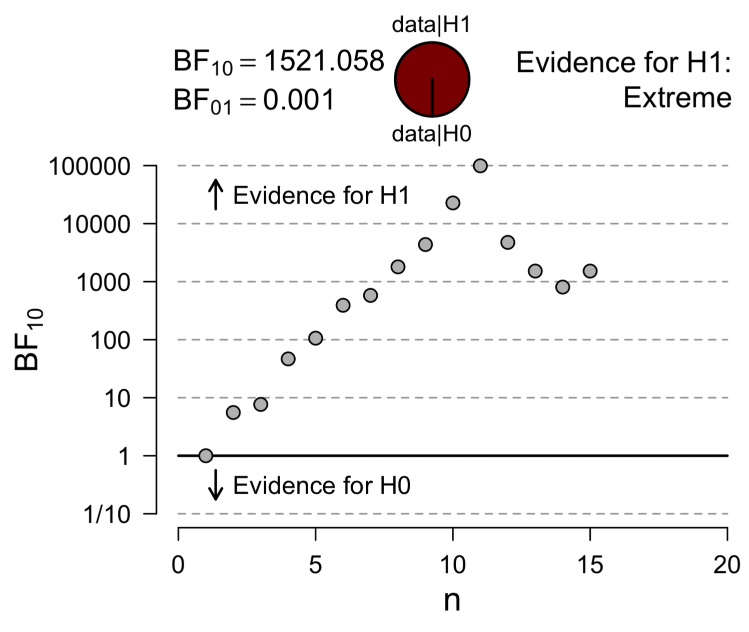

Supplement: Supplementary file 2 — Additional file 2 PDF export of JASP-file for the dementia patient data analysis [file 12874_2020_980_MOESM2_ESM.pdf]
