# Supplementary material for: Bayesian alternatives to null hypothesis significance testing in biomedical research: a non-technical introduction to Bayesian inference with JASP
Source: BMC Med Res Methodol. 2020 Jun 5;20:142. doi: 10.1186/s12874-020-00980-6 (PMC7275319; doi:10.1186/s12874-020-00980-6)

Physical Activity and BMI: Linear Regression

Description:

This data set, "Physical activity and BMI", provides participants' Body Mass Index and average daily number of steps.

Variables:

- **SUBJECT -** Participant's ID.
- **PA -** Physical activity indicated by average daily number of steps (in thousands).
- **BMI -** Body Mass Index

This example JASP file demonstrates the use of linear regression. Specifically, we will investigate whether and to what extent the average daily number of steps relates to BMI.

Reference:

Moore, D. S., McCabe, G. P., and Craig, B. A. (2012). *Introduction to the Practice of Statistics* (7th ed.). New York: Freeman.

Mestek, M. L., Plaisance, E., and Grandjean, P. (2008). The relationship between pedometer-determined and self-reported physical activity and body composition variables in college-aged men and women. *Journal of American College Health*, 57: 39–44.

Descriptive Statistics

The data feature 100 participants with recordings of their physical activity (PA) and Body Mass Index (BMI).

Descriptive Statistics

	BMI	PA
Valid	100	100
Missing	0	0
Mean	23.939	8.614
Std. Deviation	3.941	2.320
Minimum	14.200	3.186
Maximum	35.100	14.209

Correlation plot

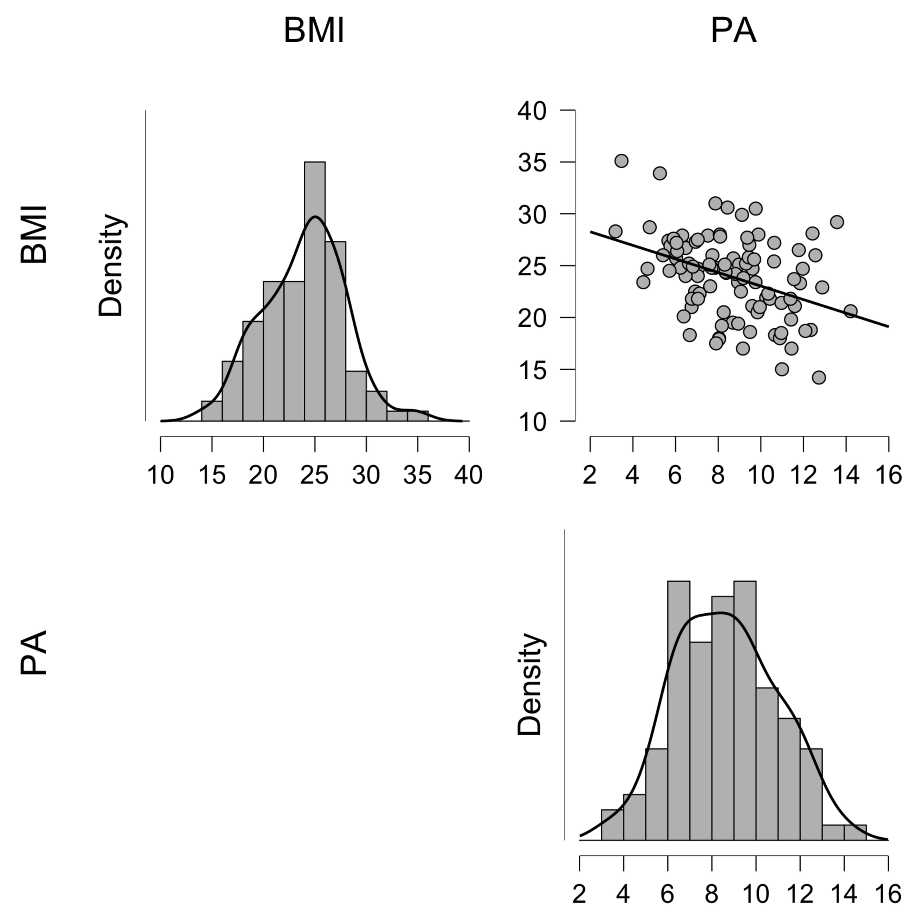

The bi-variate scatterplot shows a negative relationship between the PA and BMI.

Linear Regression

Model Summary

Model	R	R²	Adjusted R²	RMSE
1	0.385	0.149	0.140	3.655

Physical activity explains about 15% of the variance in BMI.

ANOVA

Model		Sum of Squares	df	Mean Square	F	p
1	Regression	228.377	1	228.377	17.096	< .001
	Residual	1309.101	98	13.358		
	Total	1537.478	99			

The linear regression model is significantly better than the null model.

Coefficients

Model		Unstandardized	Standard Error	Standardized	t	p
1	(Intercept)	29.578	1.412		20.948	< .001
	PA	-0.655	0.158	-0.385	-4.135	< .001

The coefficients table shows that the model can be expressed as BMI = 29.578 - 0.665 * PA.

On average, a thousand more steps per day relates to a decrease of BMI of about 0.665.

Bayesian Linear Regression

Model Comparison

Models	P(M)	P(M|data)	BF_M	BF_{10}	R²
Null model	0.500	0.004	0.004	1.000	0.000
PA	0.500	0.996	284.327	284.327	0.149

Posterior Summary

Posterior Summaries of Coefficients

						95% Credible Interval	
Coefficient	Mean	SD	P(incl)	P(incl|data)	$BF_{inclusion}$	Lower	Upper
Intercept	23.939	0.366	1.000	1.000	1.000	23.244	24.615
PA	-0.609	0.157	0.500	0.996	284.327	-0.908	-0.326

Posterior Coefficients with 95% Credible Interval

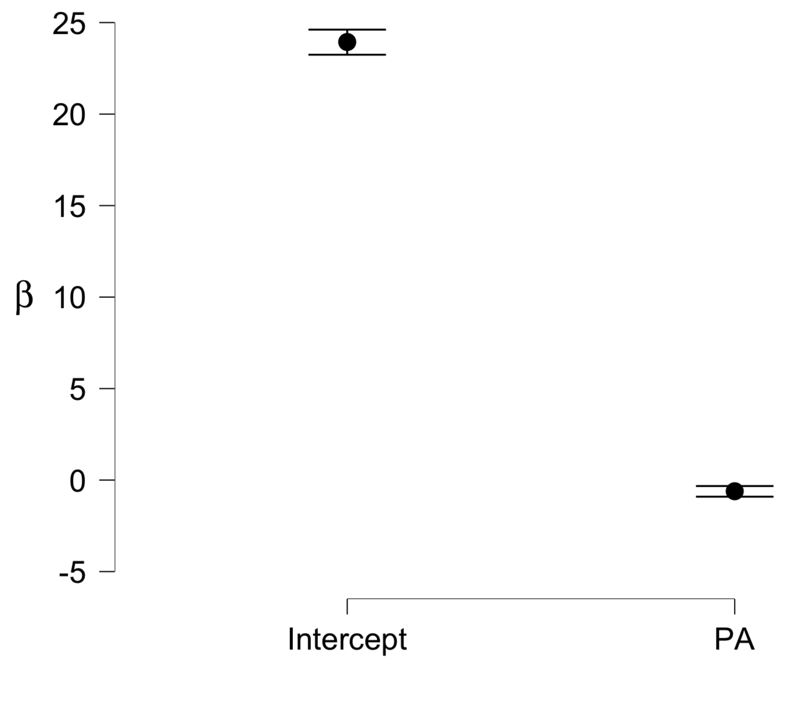

Inferential Plots

Residuals vs Fitted

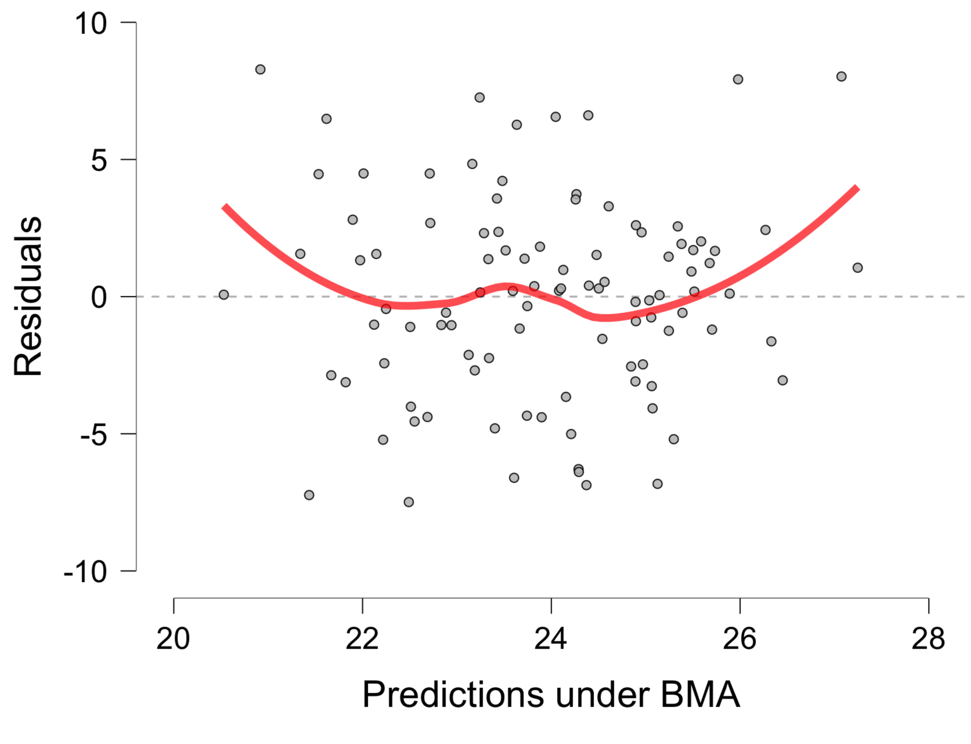

Supplement: Supplementary file 3 — Additional file 3 PDF export of JASP-file for the physical activity and BMI data analysis [file 12874_2020_980_MOESM3_ESM.pdf]
